# Object relations are processed with, but not without, awareness

**DOI:** 10.1093/nc/niaf010

**Published:** 2025-05-08

**Authors:** Shaked Palgi, Tamara Bester-Arest, Nathan Faivre, Liad Mudrik

**Affiliations:** Sagol School of Neuroscience, Tel Aviv University, PO Box 39040, Ramat Aviv, Tel Aviv 69978, Israel; Department of Brain Sciences, Weizmann Institute of Science, Herzl St 234, Rehovot 7610001, Israel; Sagol School of Neuroscience, Tel Aviv University, PO Box 39040, Ramat Aviv, Tel Aviv 69978, Israel; LPNC CNRS UMR 5105 Université Grenoble Alpes, 1251 rue des Universités, Grenoble 38058, France; Sagol School of Neuroscience, Tel Aviv University, PO Box 39040, Ramat Aviv, Tel Aviv 69978, Israel; School of Psychological Sciences, Tel Aviv University, PO Box 39040 Ramat Aviv, Tel Aviv 69978, Israel; Brain, Mind, and Consciousness Program, Canadian Institute for Advanced Research (CIFAR), Toronto, ON, Canada

**Keywords:** consciousness, unconscious processing, EEG, object relations, N400

## Abstract

The scope of unconscious integration is widely debated. Here, we examined this question, focusing specifically on deciphering the relations between two associatively related objects, in a set of five behavioral and electrophysiological experiments. Participants were presented with masked pairs of related and unrelated objects and were asked to judge their relatedness. When the masked pairs were visible, we found both a behavioral priming effect and a difference in the magnitude of the electrophysiological N400 component for unrelated compared with related pairs. In sharp contrast, when the pairs were invisible (validated using both subjective and objective awareness measures), no convincing evidence was found for relatedness processing: with electroencephalography, no difference in N400 amplitude nor above-chance decoding of pair relations was found in two separate experiments. Based on these results, we conclude that the data do not support unconscious relatedness processing, suggesting that consciousness might have a prominent role in enabling relational integration beyond the single object level, which is in line with leading theories of consciousness.

## Introduction

Perceptual consciousness and integration are considered to be closely related, both due to the unified nature of conscious percepts, and to the need for consciousness in integrative processes (Mudrik et al. 2014). Most theories of consciousness agree that conscious processing allows for integration, while differing in the mechanism they assign to consciousness [e.g. global broadcasting (Dehaene and Naccache 2001); or recurrent processing (Lamme and Roelfsema 2000); to give two examples] and in the degree of integration they asign to unconscious processing. These theories are at odds with claims that integration—and essentially any cognitive function—can take place without consciousness (Hassin 2013). The latter stand has been supported by empirical findings where integrative processes were reported to occur during unconscious processing (for reviews, see Mudrik et al. 2014, Hirschhorn et al. 2020). In such studies, the experimenters first demonstrate that participants are indeed unaware of the stimuli. They typically do so using subjective measures (where participants rate the visibility of the stimuli), objective measures (where they perform some judgment on the stimuli), or a combination of both subjective and objective measures (for review, see Seth et al. 2008). Then, they demonstrate that these unconsciously processed stimuli can be integrated.

Specifically, some studies focused on the ability to integrate between two distinct stimuli and decipher their relations (Mudrik et al. 2014); this was done, for example, with pairs of letters (Van Opstal et al. 2010), even across modalities (Faivre et al. 2014, though notably, this was only found after conscious exposure), as well as with multiple words (e.g. van Gaal et al. 2014, Scott et al. 2018).

Yet the extent of such unconscious integration is still unclear. An ongoing debate concerns higher-level integration processing, and their potential dependency on conscious processing; some studies suggested high-level integration (e.g. Mudrik et al. 2011, Ric and Muller 2012, Sklar et al. 2012) but many of them were later challenged (Shanks 2017, Moors and Hesselmann 2018) or not replicated (Moors et al. 2016, Biderman and Mudrik 2017, Rabagliati et al. 2018). Thus, the boundaries of integration without awareness—specifically when integrating multiple objects or words—remain unclear.

This ability to process the relations between two distinct visual objects is especially interesting, as it occurs regularly between seen objects, whenever we encounter a visual scene (for review, see Bar 2004). We are also great experts in processing individual objects, at least when it comes to category-level judgments; objects are relatively easily identified even when presented very briefly (e.g. Davenport and Potter 2004, Furtak et al. 2022).

Importantly for the context of this work, some studies suggest that individual objects can also be processed without awareness (for review, see Mudrik and Deouell 2022), though the results are still mixed. At the behavioral level, a few studies reported that participants can differentiate between unconsciously processed objects and animals (Dell’Acqua and Grainger 1999, Almeida et al. 2008, Van den Bussche et al. 2009a), yet this effect has not been replicated (Stein et al. 2020). Notably, however, this failure to replicate might stem from the inability to generalize the processing of pictorial stimuli to target words (since in two of the original studies, both primes and targets were pictures, while in the more recent one, the target was a word) or from too strong masking that abolished the effects. Neuropsychological studies with Neglect patients further suggested that objects can be processed even when they are presented in the patients’ blind visual field (Marshall and Halligan 1988, McGlinchey-Berroth et al. 1993; though see Bisiach and Rusconi 1990). Finally, with functional Magnetic Resonance Imaging, differential activations were found for man-made vs. natural objects embedded in scenes (Fang and He 2005). Thus, despite these conflicting results, overall it seems plausible to assume that isolated objects might indeed be processed, at least to some level, even without awareness. But does this imply that the relations between objects can also be processed unconsciously?

Thus far, this question has not been directly tested. Yet some studies imply that the answer might be positive. For example, one study probed the time it takes stimuli to emerge to awareness, which might hint on the way these stimuli are unconsciously processed (but see Stein et al. 2011). There, typically arranged pairs emerged into awareness faster than atypically arranged ones (Stein et al. 2015; though see Gronau and Shachar 2014, where spatial relations were not processed without attention).

To examine if processing the relations between two unseen objects is possible, we conducted five experiments (three of which were preregistered—linked attached in “Additional Info”) in which semantically related (e.g., a camera and a tripod) and unrelated (e.g., fireworks and chewing gum) pairs of objects were presented. In all studies, we used visual masking to manipulate stimulus visibility (Breitmeyer 2015). We assessed if related and unrelated object pairs elicited either a priming effect (i.e., facilitated processing of a visible stimulus following the presentation of a congruent invisible stimulus, compared with an incongruent one) or different electroencephalographic event-related potentials (ERPs). We specifically targeted the widely studied N400 ERP component, which is hypothesized to index semantic processing and integration (for review and alternative interpretations, see Kutas and Federmeier 2011), also between objects (e.g. Barrett and Rugg 1990, Zucker and Mudrik, 2019). We reasoned that the N400 could serve as a marker for unconscious processing of the relations between the objects, potentially providing higher sensitivity than the behavioral priming effect (cf. Fahrenfort et al. 2017 and van Gaal and Lamme 2012, where behavioral effects were not found, yet electroencephalography (EEG) patterns indicated unconscious processing).

## Experiments 1–2: behavioral evaluation through priming

### Methods

#### Participants

Experiment 1 included twelve participants (five female; aged 19–28, mean = 24.8). All were students at the California Institute of Technology and the Ecole Normale Supérieure, earning course credit as compensation. The sample size for subsequent experiments (Experiments 3 and 5) was determined based on this experiment. One additional participant was excluded for meeting one of the predetermined exclusion criteria—namely, having a target performance lower than 70% (see all criteria in Supplementary Information—Extended Data 1). The experimental procedure was approved by the IRB committee of the California Institute of Technology, and informed consent was obtained from the participants after the procedure had been explained to them.

Experiment 2 involved 25 participants (18 female; aged 19–32, mean = 24.2). They were either individuals studying at Tel Aviv University, receiving course credit as compensation, or healthy adults who took part in the study for payment. The sample size of this experiment was chosen to match the sample size of a pilot study that preceded it (see Supplementary Information—Extended Data 4). The experimental procedure was approved by the ethics committee of Tel Aviv University, and informed consent was obtained from the participants after the procedure had been explained to them.

#### Apparatus

Participants sat in a dimly lit room, and the stimuli were presented on a gray background (RGB: 128, 128, and 128), using Matlab and Psychtoolbox 3 (Pelli 1997).

In Experiment 1, participants were positioned at a distance of 57 cm from the screen and viewed the stimuli on a 19” CRT monitor operating at a 60 Hz refresh rate. The stimuli were presented at a visual angle of 6.4° (width) × 3.6° (height). In Experiment 2, they were positioned 60 cm from the screen and viewed the stimuli on a 23” CRT monitor. The stimuli were presented at a visual angle of 6.96° (width) × 4.3° (height).

#### Stimuli

**Figure 1:**
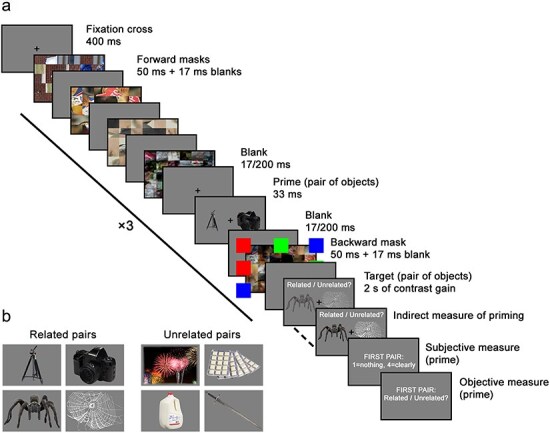
Example stimuli and trial procedure in Experiments 1–2. (a) Trial procedure for Experiments 1–2. The only difference between the conscious (Experiment 1) and the unconscious (Experiment 2) conditions was the Stimulus-Onset-Asynchrony (SOA) between the prime and following mask, which were 200 ms for the conscious condition or 17 ms for the unconscious one. (b) Example stimuli. Left: two pairs with associative relations (Related pairs). Right: two pairs without any relation (Unrelated pairs). We note that the actual stimuli used in the experiments were copyrighted and therefore replaced in this figure by equivalent images.

The stimulus bank included pairs of colored real-life images taken from Internet sources, akin to the one used in Zucker and Mudrik (2019). The images either depicted objects, people, or scenes. They were digitally pasted onto a gray background (RGB: 128, 128, and 128). Related pairs conveyed an associative relationship between the two objects (i.e., objects that tend to co-appear, like a camera and a tripod; Fig. 1b), while unrelated pairs did not convey such a relationship (e.g., fireworks and chewing gum, Fig. 1b). A full description of the stimulus bank, including controls for low-level perceptual features (brightness, chromaticity, etc.) and a validation questionnaire can be found in Zucker and Mudrik (2019). The initial set of stimuli also includes a third category (“abstract relations”) that was intentionally omitted in this study to maintain task simplicity.

The specific pairs used for each participant (50 related and 50 unrelated) were chosen at random. Each pair had two versions, using different exemplars of the same objects, persons, or scenes (e.g., the first stimulus set included a pair of a hand-mirror and a comb, and the second set included a pair of a different hand-mirror and a different comb). This second set was used for the target stimuli, such that the same exact images were not used for primes and targets.

In addition to these stimuli, masks were made by dividing other images depicting real-life scenes into 5 × 6 matrices, and then randomly shuffling the cells within each matrix.

For Experiment 2, an additional set of stimuli was used for a calibration session as well as for a post-session visibility test. A description of this stimuli-set can be found in Supplementary Information—Extended Data 2.

#### Experimental design

This experiment looked for a behavioral priming effect of object relations, by manipulating two types of relations. First, *pair relatedness*: as described above, within each prime and target pair, two images were presented, which could either be related or unrelated to each other (see again the Stimuli section and Fig. 1b). Second, *prime-target congruency*: as both the prime and the target stimuli were pairs of objects that can either be related or not, we could also manipulate the relations between the prime and the target. In congruent trials, both prime and target conveyed the same relations. In incongruent trials, if the prime was a related pair of objects, the target was an unrelated pair, and vice versa.

Thus, this was a 2-by-2 design with prime relatedness (related/unrelated pairs of images) and prime-target congruency (identical/different) as within-subject variables. Overall, the experiment included three blocks of 100 trials. In each block, half the prime pairs were related (i.e., the two images were related to one another), and half the primes were unrelated. In addition, half the trials were congruent (i.e., prime and target pairs conveying the same relations: either both prime and target depicted related images or both depict unrelated images) and half were incongruent (i.e., prime and target pairs conveying the opposite relations: one related and the other unrelated). Trials were intermixed, with the constraint that the same trial type (with respect to both prime-target congruency and target relations) was not repeated for more than four consecutive trials. The location of the two objects in the pair (i.e., right or left to the fixation) was randomly determined in each trial.

#### Procedure

Each trial began with a 400 ms fixation cross. Then, four forward masks were presented for 50 ms each, followed by 17 ms blanks. Consciousness was manipulated using the Stimulus-Onset-Asynchrony (SOA) between the prime pair and the following mask. In Experiment 1, a 200 ms SOA was used, so that the prime pair was visible, while in Experiment 2, the SOA was 17 ms. Then, the prime pair was presented in the middle of the screen (i.e., one object to the left of fixation and one object to the right) for 33 ms, followed by an additional blank of 200 ms (Experiment 1) or 17 ms (Experiment 2). Finally, one backward mask was presented for 50 ms, followed again by a 17 ms blank (sandwich-masking; Breitmeyer 2015). The backward mask was accompanied by six color squares (two blue, two green, and two red), overlapping its borders and extending outwards, to enhance masking effectiveness (Mudrik and Koch 2013, Biderman and Mudrik 2017).

This entire sequence was presented three times (Macknik and Livingstone 1998) and was then immediately followed by the target pair. The target contrast was ramped up linearly from 0 to 100% within 2 s and was presented simultaneously with the question “related/unrelated?” (Q1). Accordingly, participants were able to respond from the very first moment the target was ramped up. The ramping-up procedure was introduced to make the task more sensitive to the priming effects, as during the initial ramping-up stages, when the upcoming information is still impoverished, observers are held to be more influenced by top-down processes when interpreting the upcoming information (Bar 2004, Melloni et al. 2011). The question remained on the screen for 3 s in case the participants did not respond during the 2 s period of target display (the target and the question disappeared as soon as the participants responded).

Participants responded by pressing either the right or the left arrow key with their right hand (keys assignment to responses was randomly determined for each participant, hence counterbalanced across the experiment). Then, they were asked to subjectively rate the visibility of the prime pair with their left hand on a 4-point perception awareness scale (PAS, Ramsøy and Overgaard 2004; 1 = “I didn’t see anything”; 2 = “I had a vague perception”; 3 = “I saw a clear part of the pair”; 4 = “I saw the pair clearly”; Q2). Finally, participants were asked to determine whether items in the prime pair were related or not, by again pressing either the right or the left arrow keys with their right hand (Q3). Participants continued to the next trial at their own pace. The sequence is plotted in Fig. 1a.

In Experiment 2, in addition to the main experimental session, we included two additional sessions—one for calibrating the contrast of the prime and the masks and another for testing the visibility of the primes. In these sessions, all pairs were unrelated, half of them were presented upright and the other half were inverted (pseudo-randomly intermixed, with the same constraint as in the main experimental session). Q1 was respectively replaced here with “upright/inverted?”

The calibration session included 70 trials in which different prime pairs were presented. Participants were asked to first report the orientation of the prime (and, if needed, guess) and then rate its visibility using the PAS scale described in Experiment 1.

Based on participants’ performance in the orientation task, prime and mask contrast were determined for each participant using a one-up one-down staircase procedure (Levitt 1971), such that for every correct response, the visibility conditions became harder, while for every incorrect response, they became easier. Starting from 0.85 contrast for masks and 0.7 for primes, mask contrast was first manipulated (i.e., made greater for a correct response, and smaller for an incorrect one), and if it reached 1, prime contrast was manipulated (now, made smaller for a correct response, and greater for an incorrect one), with a lower bound of 0.4. The resulting values were then used in the main experiment.

Finally, the visibility post-test was also composed of 70 trials. It was designed to complement the objective measure we used in the main experimental session and allow a more stringent assessment of visibility. In these sessions, the key question about the prime appeared immediately after it, to avoid memory interference, and it also pertained to its orientation rather than to the relations between the images, so even having partial awareness of one of the images should allow participants to respond correctly.

#### Statistical analysis

##### Participant discrimination analysis

We selected trials for analysis based on a subjective visibility criterion. In Experiment 1, which was designed to assess conscious processing, we selected only trials that were rated as visible, while in Experiment 2, which was designed to probe unconscious processing, we selected only trials that were rated as invisible. Subjective visibility assessment was based on the PAS score given by the participant in each trial. Trials marked with PAS scores 3–4 were considered visible, while trials marked with PAS score 1 were considered invisible.

In Experiment 2, probing unconscious processing, two objective measures (relatedness judgment in the main session and orientation judgment in the post-session visibility test) further validated participants’ subjective reports. Awareness was assessed in both tasks by calculating *d*ʹ of individual participants (Green and Swets 1966).

Note that the practice of participants’ exclusion was criticized for potentially evoking regression to the mean (Shanks 2017). Since in our case no effect was found, this criticism does not apply.

##### Priming analysis

Priming effects were calculated based on participants’ reaction times to Q1 (target relation). These data were first cleaned, by excluding incorrect trials (*M* = 7.9%, SD = 7.3 in Experiment 1; *M* = 9.1%, SD = 3.5 in Experiment 2), as well as trials with reaction times shorter than 200 ms or longer than 4 s (*M* = 5%, SD = 8.4 in Experiment 1; *M* = 0.6%, SD = 1.4 in Experiment 2). Then, a subset of the trials was selected for analysis based on their visibility scores.

Reaction times in the target task were analyzed using linear mixed-effects regressions, with Target relation and Prime-target congruency as fixed effects, intercepts for participants as random effects, and a by-participant random slope for the effect of relation. Model selection for additional random slopes was based on penalized likelihood estimations. All models were fitted using restricted maximum likelihood, and *P*-values were obtained by using the Satterthwaite method for approximation of degrees of freedom. Null effects were assessed using a JZS Bayes factor (BF) *t*-test with default prior scales and were interpreted using the following convention: a BF < 0.1 was taken to imply substantial evidence for an absence of effect (H0), 0.1 < BF < 0.33 indexes moderate evidence for an absence of effect, 0.33 < BF < 3 suggests insensitivity of the data, 3 < BF < 10 represents moderate evidence for an effect (H1), and BF > 10 implies substantial evidence for an effect (Lee and Wagenmakers 2014). This convention for interpreting BFs was used throughout this manuscript. All analyses were performed with R (R Core Team 2022), including the lmerTest (Kuznetsova et al. 2017) and BayesFactor (Morey and Rouder 2018) packages.

##### Correction for multiple comparisons

In order to control for multiple comparisons across the entire manuscript, we opted for a manuscript-wise procedure, as suggested in Benjamini et al. (2001). We therefore controlled the false discovery rate (FDR) across the entire manuscript, by applying the Benjamini–Hochberg procedure (Benjamini and Hochberg 1995) to all *P*-values in the manuscript. We included in this procedure all experimental results that are relevant to our research questions (i.e., not including behavioral visibility measures; *n* = 31 *P*-values across the five experiments and the two combined-datasets analyses). This allowed us to enforce a reasonable significance criterion for the experimental findings, without inflating type-1 error due to the inclusion of multiple experiments. All *P*-values reported in the manuscript are the corrected ones.

### Results

#### Experiment 1

**Figure 2:**
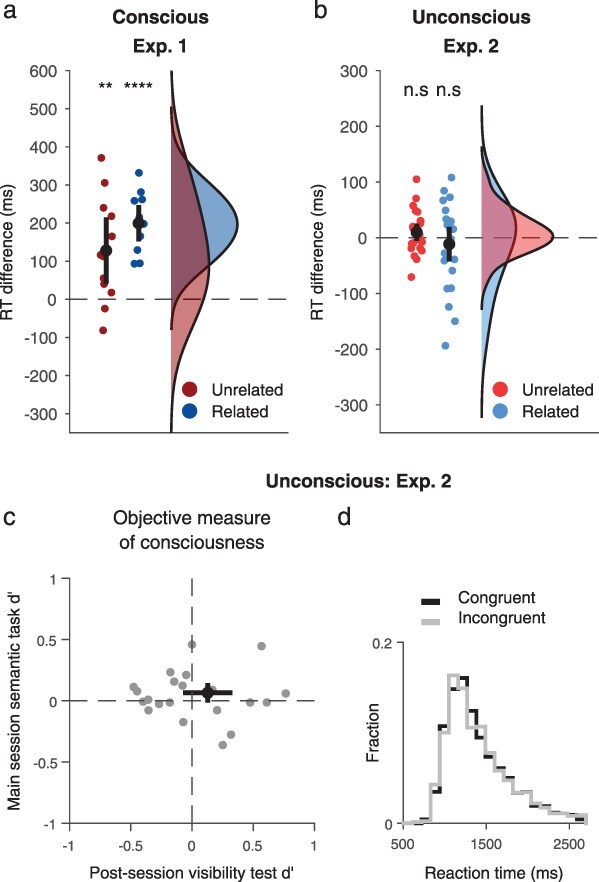
Results of Experiments 1–2. (a) Results of Experiment 1, probing conscious processing. Mean priming effect, calculated by subtracting reaction times (RTs) in congruent trials from incongruent trials. Data are plotted separately for Related targets (dark red) and Unrelated targets (dark blue). Dots correspond to individual participants (*n* = 12), with black dots and lines showing the population means and CIs. (b) Results of Experiment 2, testing unconscious processing (*n* = 25 participants). Here, lighter colors denote unconscious processing (as opposed to conscious processing presented in panel A). (c–d) Control analyses for Experiment 2. (c) Objective measure of consciousness. Individual participant *d*ʹ values for the main session semantic task (*Y*-axis) vs. the post-session visibility test (*X*-axis). Black dots and lines correspond to the population means and CIs. (d) Distribution of reaction times (ms) for congruent trials (black) and incongruent trials (gray).

Experiment 1 measured priming effects induced by related and unrelated prime pairs that participants consciously saw. Overall performance for determining if the images in the target pair were related was 92.1% (SD = 7.3%). As expected, participants rated the prime pairs as visible in 98.9 ± 1.9% of the trials (mean ± SD; visibility 1: 1.1 ± 2%, visibility 2: 2.6 ± 3.8%, visibility 3: 15.6 ± 28.7%, visibility 4: 80.8 ± 29.2%) and were accordingly very good also in determining whether images in the prime pairs were related to each other or not (*M* = 92.1%, SD = 6.7%; *M d*ʹ = 3.16, SD = 0.87, *t*(11) = 12.58, *P* < .001, 95% CI = [2.61, 3.71], BF > 10^5^). Linear mixed-effects regressions of reaction times revealed a main effect for Prime-target congruency (Fig. 2a), so that participants were overall faster in congruent trials (mean = 1419 ms, SD = 335 ms), compared to incongruent trials (*M* = 1583 ms, SD = 404 ms; *F*(1, 2815) = 90.4, *P* < .001, 95% CI = [−242, −150], BF > 10^17^). In addition, a marginal interaction between Prime-target congruency and Target relations was found (*F*(1, 2820) = 5.5, *P* = .052, BF = 0.88); *post-hoc* comparisons revealed that the effect was stronger for related pairs (*M*_diff_ = 200 ms, SD = 75 ms, *t*(11) = 9.26, *P* < .001, 95% CI = [−247, −152], BF > 10^4^) than for unrelated pairs (*M*_diff_ = 128 ms, SD = 137 ms, *t*(11) = 3.25, *P* = .027, 95% CI = [−215, −41], BF = 7.2).

#### Experiment 2

Here, unconscious processing of the prime pair was tested (Fig. 2b and 2c). We first verified that overall performance on the target was high (91.3%, SD = 5%). We then focused on the visibility of the prime pair: subjectively, most trials were rated as invisible (visibility 1: 79.7 ± 17.1%, visibility 2: 16.9 ± 13.7%, visibility 3: 2.7 ± 4.2%, and visibility 4: 0.69 ± 1.7%). Related and Unrelated trials were classified as not seen in similar proportions (Related: 79.6 ± 17.3%, Unrelated: 79.9 ± 17.2%; *t*(24) = −0.36, *P* = .72). Importantly, performance for the relatedness of the prime pairs in these trials was at chance, both for the main session (*M* = 50.9%, SD = 3.5%; *M d*ʹ = 0.06, SD = 0.19; *t*(24) = 1.69, *P* = .10, 95% CI = [−0.014, 0.14], BF = 0.73) and for the post-session visibility test (*M* = 50.4%, SD = 8.9%; *M d’* = 0.13, SD = 0.48; *t*(24) = 1.30, *P* = .20, 95% CI = [−0.073, 0.33], BF = 0.49). Crucially, participants responded at equal speed in congruent (mean = 1415 ms, SD = 203 ms) and incongruent trials (1414 ms, SD = 202 ms). Accordingly, no main effect of Prime-target congruency was found (i.e., no priming effect: *F*(1, 5143)< 0.001, *P* > .99, 95% CI = [−35, 17], BF = 0.029), nor an interaction between Prime-target congruency and Target relatedness (*F*(1, 5145) = 0.92, *P* = .50, BF = 0.073). In a complementary analysis, we also did not observe any effects of congruency on participants’ accuracy (Supplementary Information— Extended Data 5).

Given these null results, we tested if an effect might be found when using only trials where reaction times were relatively short, following previous studies that found a similar phenomenon (Avneon and Lamy 2018, 2019). We accordingly conducted an exploratory follow-up analysis, where we only included the fastest half of the trials, selected separately for each participant and condition (congruent and incongruent), and used the same mixed model as before. Again, we did not find a significant effect for Prime-target congruency (*F*(1, 2541) = 2.81, *P* = .19, BF = 0.15), and only a marginal interaction between Target relations and Prime-target congruency (*F*(1, 2533) = 5.2, *P* = .059, though BF = 0.77). To test if this marginal interaction hints at an effect, we conducted *post-hoc* tests for the effect of congruency within each relations condition, and no significant priming effects were found: Related: *t*(24) = 1.8, *P* = .18, BF = 0.89; Unrelated: *t*(24) = 0.22, *P* = .89, BF = 0.21. Finally, to more robustly test the differences between conscious and unconscious processing, and directly compare them, we conducted an exploratory analysis where we combined the two datasets of Experiments 1 and 2. We fitted a linear model that includes the Experimental condition and tested the interaction between Prime-target congruency and Experimental condition. Doing so, with an effective sample size of 37 participants, we found a significant 3-way interaction between Experimental condition, Prime-target congruency, and Target relations (*F*(1, 7966) = 7.68, *P* = .021, BF = 2.22), and—most importantly for our purposes—a 2-way interaction between Experimental condition and Prime-target congruency (*F*(1, 7958) = 82.6, *P* < 10^−15^, BF > 10^15^). This interaction reinforces the above finding, where an effect was found in the conscious experiment, and not in the unconscious one.

## Experiments 3–5—evaluating processing using EEG markers

The null behavioral result obtained in Experiment 2 might be taken as evidence for the claim that relations between two images cannot be processed without awareness. Alternatively, it could be explained by the possible insensitivity of the priming method used to probe unconscious processing. Specifically, our decision to ramp up the target pair, originally aimed at making the measure of unconscious processing more sensitive, could have acted against us, as it prolonged the time between the offset of the prime and the time at which participants started seeing the stimulus. Given that unconscious processes are typically short-lived (Greenwald et al. 1996, Avneon and Lamy 2018, 2019), this could have rendered our design less sensitive to such effects, as opposed to our original plan.

Even irrespective of that potential caveat, we reasoned that using a neural measure, specifically EEG, might provide us with a stronger tool for examining the research question. Some studies that failed to find behavioral effects were nevertheless able to demonstrate unconscious processing by comparing EEG waveforms elicited by the stimuli of interest (van Gaal and Lamme 2012, Fahrenfort et al. 2017). Thus, we ran a set of three EEG experiments to look for underlying differential neural activity that might serve as a signature of the unconscious processing of relations. These experiments were similar to Experiments 1 and 2, with slight changes to the paradigm (see Procedure description below). Experiment 4 was a conceptual replication of Experiment 3, yet with increased power; specifically, we doubled the number of trials, increased the number of participants, and selected the strongest pairs in our stimulus bank, to maximize the chances of obtaining an effect.

### Methods

#### Participants

Experiment 3 involved 24 participants (18 females; aged 19–30, mean = 23.3). They were either individuals studying at Tel Aviv University, receiving course credit as compensation, or healthy adults who took part in the study for payment. This was an exploratory experiment seeking unconscious effect and its sample size was chosen to double the sample size of Experiment 1 (which searched for conscious effect). Three additional participants were excluded for meeting the exclusion criteria (see Supplementary Information—Extended Data 1).

Experiment 4 included 45 participants (22 women; aged 18–32, mean = 24.5). They were either individuals studying at Tel Aviv University, receiving course credit as compensation, or healthy adults who took part in the study for payment. This experiment was a replication of Experiment 3, and its sample size was determined using a power analysis to allow a power of 0.9 with an effect size of 0.2. Two additional participants were excluded for meeting the exclusion criteria.

Experiment 5 had 12 participants (7 females; aged 19–27, mean = 23.7). Like in the other EEG experiments, they were either individuals studying at Tel Aviv University, receiving course credit as compensation, or healthy adults who took part in the study for payment. This was an exploratory experiment testing if an effect can be found during conscious processing and its sample size was chosen to match the sample size of Experiment 1 (which focused on conscious processing as well). One additional participant was excluded for meeting exclusion criteria.

In all three experiments, the experimental procedure was approved by the ethics committee of Tel Aviv University, and informed consent was obtained from the participants after the procedure had been explained to them.

#### Apparatus

The same apparatus as in Experiment 2 was used, but the monitor operated at a 100 Hz refresh rate, and the stimuli were presented at a visual angle of 6.96° (width) × 4.3° (height).

#### Stimuli

The same stimuli as depicted in Experiments 1–2 were used here. In Experiment 3, they were used without any modification (50 related pairs and 50 unrelated pairs, randomly chosen). In Experiments 4 and 5, we changed a few details to maximize the chances of finding an effect: First, we sub-selected specific stimuli from the whole set: We used 55 pairs that were previously rated as most strongly related , and 55 that were rated as most unrelated. For the chosen related pairs, the accuracy in judging them as related was high (*M* = 97.6%, SD = 3.2%), and their relation was rated as strong (*M* = 4.6, SD = 0.2, on a scale of 1–5 where 5 being highly related). For the chosen unrelated pairs, accuracy in judging them as unrelated was again high (*M* = 94%, SD = 4.9%). These numbers were taken from the validation questionnaire of the original stimuli set (Zucker and Mudrik 2019). In addition, whereas in Experiments 1–3, a single image could appear in more than one pair (e.g., once with a related object and once with an unrelated one), the subset of pairs chosen for Experiments 4 and 5 did not include any semantic repetition between pairs—each image appeared only within a single pair.

#### Experimental design

This experiment focused on finding EEG markers of objects integration. Thus, only one pair of objects was presented in each trial, with pair congruency being a single within-subjects independent variable. Experiments 3 and 4 were designed to assess unconscious processing (akin to Experiment 2), while Experiment 5 was designed to be their conscious counterpart (similar to Experiment 1).

#### Procedure

##### Experiment 3

The main session included two blocks, each with 100 trials. Related and unrelated object-pairs were divided equally in each block and were presented in a pseudo-random order. Each unique pair was repeated once in each block, but with different exemplars.

A similar masking approach as in the previous experiments was used (sandwich-masking). Here, however, behavioral priming was not assessed. Instead, emphasis was placed on the EEG responses elicited by a pair of objects, which could either be related or unrelated.

**Figure 3:**
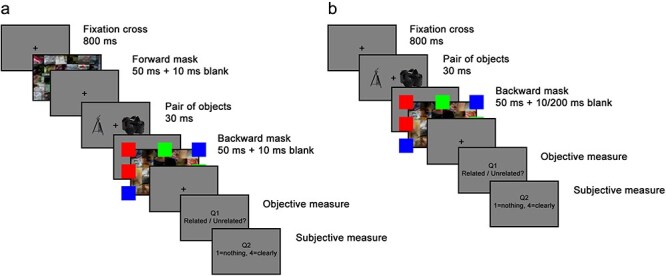
Trial procedures of Experiments 3–5. (a) Trial procedure for Experiment 3. (b) Trial procedure for Experiments 4–5. Akin to Experiments 1–2, the only difference between the unconscious (Experiment 4) and the conscious (Experiment 5) conditions was the duration of the first blank after the target, which was 200 ms for the conscious condition or 10 ms for the unconscious one.

Accordingly, each trial featured only one masked pair of objects. Additionally, a single forward mask, not four, was used, and the stimulation sequence was presented only once instead of being repeated three times. This was done in order to minimize the number of events in each trial and get a cleaner EEG signal.

Each trial began with an 800 ms fixation cross. Then, one forward mask was presented for 50 ms, followed by a 10 ms blank. The pair of objects was subsequently presented for 30 ms, followed by a 10 ms blank. Finally, one backward mask was presented for 50 ms, followed again by a 10 ms blank. The backward mask was the same as in the previous experiments.

This sequence was immediately followed by a question “related/unrelated?” (Q1), which remained on the screen until the participant responded , or for 3 s, if no response was given by then. Participants responded by pressing either the right or the left arrow keys with their right hand. Then, they were asked to subjectively rate the visibility of the pair with their left hand, using the PAS metric described in Experiment 1 (Q2). The entire sequence is depicted in Fig. 3a.

Akin to Experiment 2, the main experimental session was preceded by a pre-session calibration block (composed of 100 trials) and followed by a post-session visibility test (composed of 200 trials, divided into 2 blocks; number of trials was increased here in an attempt to improve the reliability of this measure; (Yaron et al., 2024). Again, all pairs were unrelated, some upright and some inverted and Q1 addressed the orientation of the objects. The presentation sequence in these sessions matched the one used in the main experimental sessions.

##### Experiments 4 and 5

In order to increase the chance to find an effect, a few changes were made from the procedure of Experiment 3: first, 10 additional trials were added to the main experimental session, increasing the number of trials in this session to 110 (keeping them still half related and half unrelated).

In addition, in these experiments, we opted for a weaker masking scheme, using only backward masking with no forward masks blank (backward-masking, Breitmeyer 2015). Each trial began with an 800 ms fixation cross. Then, the pair of objects was presented for 30 ms, followed by a blank of 10 or 200 ms (for Experiments 4 and 5, respectively). Then, a backward mask was presented for 50 ms followed again by a 10 ms blank. The backward mask was the same as in all previous experiments. This backward masking paradigm was used both in the main session and in the calibration and visibility test blocks. The experimental sequence is depicted in Fig. 3b. In addition, in these experiments, we also counterbalanced the response keys assignment (related/unrelated) across participants, such that for half of them, the right arrow indexed the related response (and the left—the unrelated response), and for the other half, vice versa.

#### Participant discrimination analysis

Similar to Experiments 1 and 2, we selected trials for analysis based on subjective responses on the PAS scale. In Experiment 3, only invisible trials were selected (PAS score 1), and in Experiment 5, only visible trials were selected (PAS scores 3–4).

In Experiment 4, we decided to slightly loosen our criteria for selecting invisible trials, to increase the chances of finding an effect in favor of unconscious processing. We, therefore, adopted a procedure suggested by Hesselmann et al. (2016): in brief, if a participant had <20 valid visibility-1 trials (i.e., trials marked with a PAS score of 1) in one of the conditions (Related/Unrelated), and performance was still at chance level in both the main experimental session *and* the visibility-test session when including also visibility-2 trials (assessed using a binomial test; three participants were included using this criterion), these trials were also included. If performance was above chance, the participant was excluded from the dataset and we ran an additional participant.

#### EEG recording

EEG activity was recorded using an Active 2 system (BioSemi, the Netherlands) from 64 electrodes distributed based on the extended 10–20 system connected to a cap and additional external electrodes (7 in Experiment 3 and 6 in Experiments 4 and 5). Four of the external electrodes recorded the electrooculogram (EOG) channels: two located at the outer canthi of the right and left eyes and two above and below the center of the right eye. Two external electrodes were located on the mastoids to be used as reference. All electrodes were referenced during recording to a common-mode signal electrode that was located between electrodes POz and PO3. The EEG was continuously sampled at 512 Hz, with an online high pass filter of 0.05 Hz, and was stored for offline analysis. Electrode impedances were kept below 30 kV.

#### Preprocessing and data segmentation

Preprocessing was conducted using the “Brain Vision Analyzer” software (Brain Products, Germany). For consistency with previous N400 studies (Kutas and Federmeier 2011, Zucker and Mudrik 2019), data from all channels were referenced offline to the average of the mastoid channels. The data were digitally high-pass filtered at 0.1 Hz (24 dB/octave) to remove slow drifts, using a Butterworth zero-shift filter, and then digitally notch filtered at 50 Hz to remove electrical noise. Bipolar EOG channels were calculated by subtracting the left from the right horizontal EOG channel, and the inferior from the superior vertical EOG channels, to emphasize horizontal and vertical eye movement artifacts. The signal was then cleaned of blink and saccade artifacts using Independent Component Analysis (ICA, Jung et al. 2000). Next, we used a semi-automatic artifact removal procedure to detect and remove segments with artifacts (amplitudes exceeding 100 mV, differences beyond 100 mV within a 200 ms interval, or activity below 0.5 mV for over 100 ms). Specific channels with high rejection rates on valid trials were interpolated from the other electrodes using splines (Order: 4, Degree: 10, Lambda: 10^–5^) resulting in one participant having three electrodes interpolated in Experiment 3, and one participant having one electrode interpolated in Experiment 4.

The EEG data were segmented into 1000 ms long epochs starting 200 ms prior to the stimulus. To correct for drifts, we ran a baseline correction procedure. We first calculated the mean of the first 100 ms of each segment per participant (i.e. −200 to −100 ms) and then averaged those values and subtracted the resulting value from each time point in all segments. Next, the segments were averaged by condition (Experiment 3: related: *n* = 58.42 ± 18.34 trials, unrelated: *n* = 59.08 ± 18.62 trials; Experiment 4: related: *n* = 64.29 ± 22.68 trials, unrelated: *n* = 69.22 ± 20.47 trials; Experiment 5: related: *n* = 66.17 ± 12.62 trials, unrelated: *n* = 43.50 ± 12.40 trials). The average waveforms were low-pass filtered using a Butterworth zero-shift filter with a cutoff of 30 Hz. For visual purposes only, we further low-pass filtered the signal using a Butterworth zero-shift filter with a cutoff of 12 Hz. To reduce the number of comparisons, electrode data were pooled to nine electrode groups, defined by their location (Mudrik et al. 2010): Left, Middle, Right × Frontal, Central, Parieto-Occipital (Left Frontal [Fp1, AF3, AF7, F3, F5, F7]; Middle Frontal [Fpz, AFz, Fz, F1, F2]; Right Frontal [Fp2, AF4, AF8, F4, F6, F8]; Left Central [FC3, FC5, FT7, C3, C5, T7, CP3, CP5, TP7]; Middle Central [FCz, FC1, FC2, Cz, C1, C2, CPz, CP1, CP2]; Right Central [FC4, FC6, FT8, C4, C6, T8, CP4, CP6, TP8]; Left Parieto-Occipital [P3, P5, P7, P9, PO3, PO7, O1]; Middle Parieto-Occipital [Pz, P1, P2, POz, Oz, Iz]; and Right Parieto-Occipital [P4, P6, P8, P10, PO4, PO8, O2]).

#### N400 analysis

For the N400 analysis, the average amplitude of the pooled ERPs within a predefined window of 300–500 ms was used (Zucker and Mudrik 2019). This was then analyzed by a three-way repeated measures ANOVA with Region (Frontal, Central, and Parieto-occipital), Laterality (Left, Midline, and Right), and Relation type (Related and Unrelated) as factors. Greenhouse and Geisser correction was used where needed (uncorrected degrees of freedom are reported). The use of ANOVA was chosen to be consistent with previous work using this same stimuli dataset (Zucker and Mudrik 2019). Notably, when tested using Linear Mixed Models, the results remained largely the same (see Supplementary Information—Extended Data 7). This statistical approach was supplemented by a Bayesian analysis, to further assess the meaning of a null effect. The same three-way design was used, with default prior, focusing on all models containing “Relation” as a factor. All analyses were preregistered and conducted using JASP (JASP Team 2021).

#### EEG decoding

In addition to the ANOVA, we opted for a decoding approach, as suggested by Bae and Luck (2018). Briefly, the idea is to predict the relation type of each trial from the EEG signal, potentially discovering effects that cannot be seen with classical univariate analyses. To that end, trials from the same condition (related and unrelated) were randomly divided into 20 groups, ten from each condition, and then averaged (i.e., creating 20 pseudotrials). We then trained and tested Support Vector Machine (SVM) classifiers using a 10-fold cross-validation: each time we trained the classifiers using 90% of the data (18 averaged-trials) and tested their performance on the remaining two. The SVM classifiers were trained separately on the data from each time point (256 separate classifiers, with the 64 electrodes as features). The random division and prediction were repeated 50 times, to calculate the accuracy of the decoder. Finally, the decoding accuracy per participant was smoothed using a 5-point moving average. Notably, since the decoding is performed on aggregated trials, the two conditions were balanced in training and test (although the number of single-trials that were averaged was not always equal for every participant, as only trials marked with visibility 1 were used). Statistical significance at each time point was assessed using a one-tailed *t*-test of the per-participant accuracy against chance (50%). We used one-tailed statistics here, as the SVM approach we adopted could not yield meaningful below-chance decoding (Bae and Luck 2018), but we note that the results remain quantitatively the same when using two-tailed tests.

### Results

#### Experiment 3

Again, we only used trials in which participants declared not seeing the stimulus at all (visibility 1 trials: 72.1 ± 21.3% of the trials; visibility 2: 22.8 ± 17.6%; visibility 3: 4.6 ± 6.6%; and visibility 4: 0.5 ± 1.9%). As in the previous experiments, performance was at chance level for the relatedness judgment (*M* = 49%, SD = 3.7%; *M d*ʹ = 0.01, SD = 0.2, *t*(23) = 0.34, *P* = .74, 95% CI = [−0.07, 0.1], BF = 0.23; see also Supplementary Information - Extended Data 5 and Supplementary Fig. 2), as well as for the orientation judgment in the post-session visibility test (*M* = 50.9%, SD = 3.1%; *M d*ʹ= −0.09, SD = 0.29, *t*(23) = −1.54, *P* = .138, 95% CI = [−0.22, 0.03], BF = 0.6).

**Figure 4:**
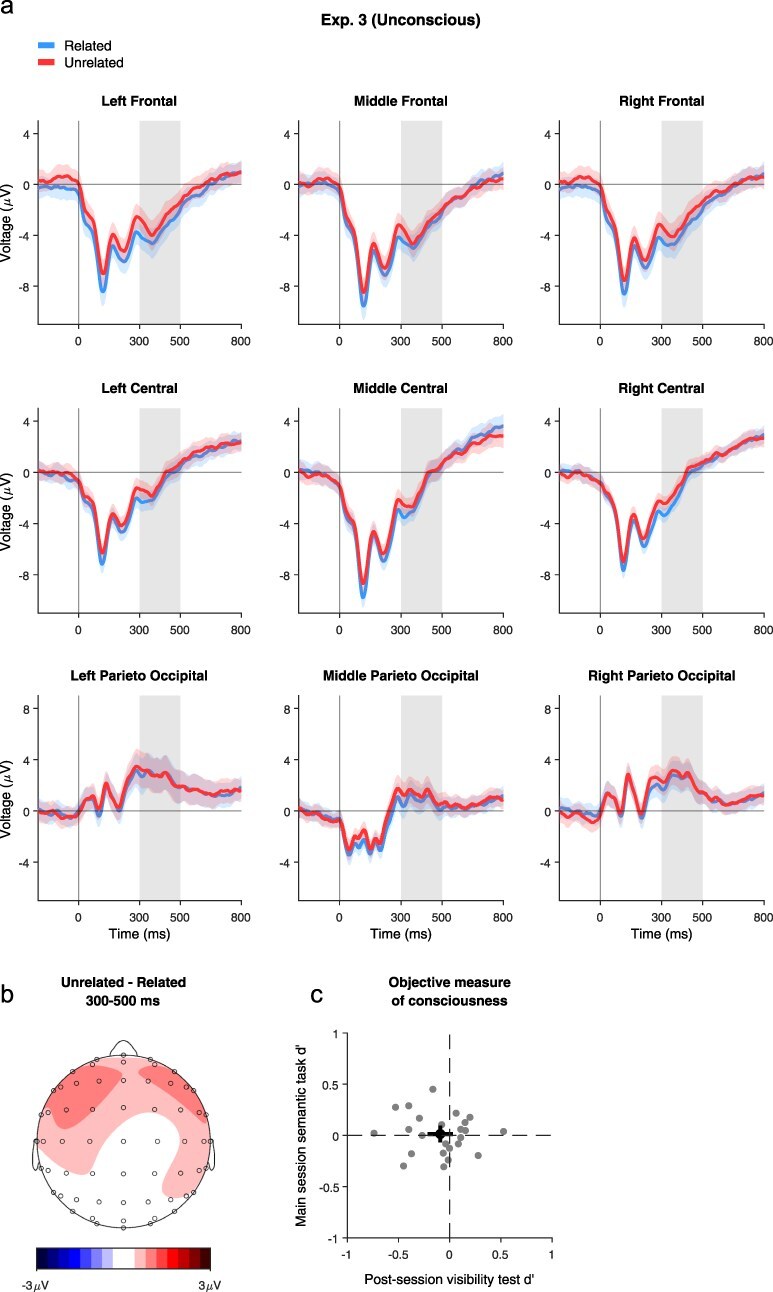
Results of Experiment 3. (a) Averaged waveforms from Experiment 3, locked to stimulus onset, for each condition and electrode group. Blue: Related pairs; Red: Unrelated pairs. The shaded area denotes the adjusted within-participant confidence intervals (Cousineau 2005, Morey 2008). Gray patches correspond to the analyzed N400 time window. (b) Topographic map of the difference wave (Unrelated–Related) at the N400 time window. (c) Objective measure of consciousness. Individual participant *d*ʹ values (*n* = 24) for the main session semantic task (*Y*-axis) vs. the post-session visibility test (*X*-axis). Black dots and lines correspond to the population means and CIs.

The three-way repeated measures ANOVA on the averaged EEG signal during the 300–500 time window in the defined areas did not yield any evidence for differential processing of related and unrelated pairs (Fig. 4), and this was further confirmed by the Bayesian analysis: no main effect of Relatedness (*F*(1, 23) = 1.33, *P* = .42, BF = 0.62) and no interaction between Relatedness and Region (*F*(2, 46) = 0.18, *P* = .78, BF = 0.062), between Relatedness and Laterality (*F*(2, 46) = 0.16, *P* = .89, BF = 0.047), or between Relatedness, Region, and Laterality (*F*(4, 92) = 1.33, *P* = .42, BF = 0.034).

#### Experiment 4

Thus far, the data seem to indicate that consciousness is needed for processing the relations between image pairs. Yet, one could still claim that our masking procedure might have reduced the signal to such a degree that it would not allow any form of processing. Thus, in Experiment 4, we aimed at maximizing the chances of finding an unconscious effect, if such an effect exists, by using only backward masking, focusing only on pairs in which the images were most strongly related/unrelated to each other, and increasing the sample size.

Here, due to the weaker masking procedure, only 67% of the trials were rated as invisible (visibility 1: 62.3 ± 24.6%; visibility 2: 26.4 ± 17.7%; visibility 3: 9.7 ± 10.7%; and visibility 4: 1.6 ± 2.7%). Interestingly, despite showing low accuracy, *d*ʹ was slightly but significantly higher than zero for the relatedness judgment (*M* = 49%, SD = 4.6%; *M d*ʹ = 0.12, SD = 0.24, *t*(44) = 3.23, *P* = .002, 95% CI = [0.04, 0.19], BF = 13.9; see Supplementary Information—Extended Data 8), but not in the non-semantic post-session visibility test, where participants were only asked to determine if the images were presented upright or inverted (*M* = 50.7%, SD = 5.3%; *M d*ʹ = −0.004, SD = 0.2, *t*(44) = −0.13, *P* = .895, 95% CI = [−0.07, 0.06], BF = 0.16).

**Figure 5:**
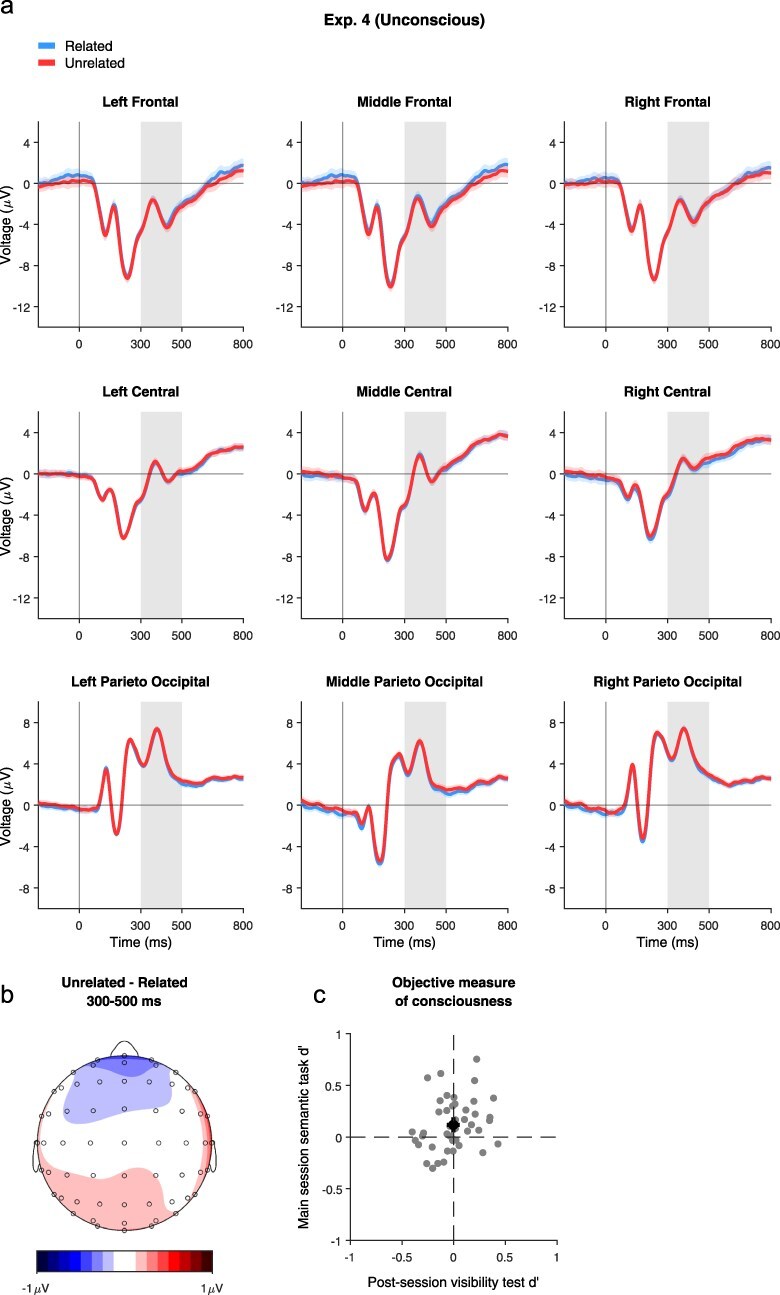
Results of Experiment 4. (a) Averaged waveforms from Experiment 4, locked to stimulus onset, for each condition and electrode group. The same conventions as in Fig. 4 are used here. (b) Topographic map of the effect of Relatedness (Unrelated–Related) at the N400 time window. (c) Objective measure of consciousness. Individual participant *d*ʹ values (*n* = 45) for the main session semantic task (*Y*-axis) vs. the post-session visibility test (*X*-axis).

Most importantly, an ANOVA on ERP amplitudes revealed no effect of Relatedness (congruency main effect = *F*(1, 44) = 0.031, *P* = .89, BF = 0.082; interaction of Relatedness and Region: *F*(2, 88) = 1.49, *P* = .42, BF = 0.038; Relatedness and Laterality: *F*(2, 88) = 0.69, *P* = .55, BF = 0.028; relatedness × Region × Laterality: *F*(4, 176) = 1.38, *P* = .42, BF = 0.013; Fig. 5), all showing conclusive evidence for the null hypothesis. Despite the higher power, the weaker masking, and the stronger pairs used, we conclude that the unconscious processing of prime pairs did not take place in Experiment 4.

#### Experiment 5

To make sure the null results found in Experiments 3 and 4 are meaningful and do not simply stem from limitations of our procedure (specifically, perhaps the relations between the images cannot be processed at all when they are presented for 30 ms only, irrespective of consciousness), we conducted a final experiment in which the stimuli were consciously perceived despite being presented for the same duration of 30 ms. This was achieved by prolonging the blank between the pair and the subsequent mask from 10 to 200 ms.

**Figure 6:**
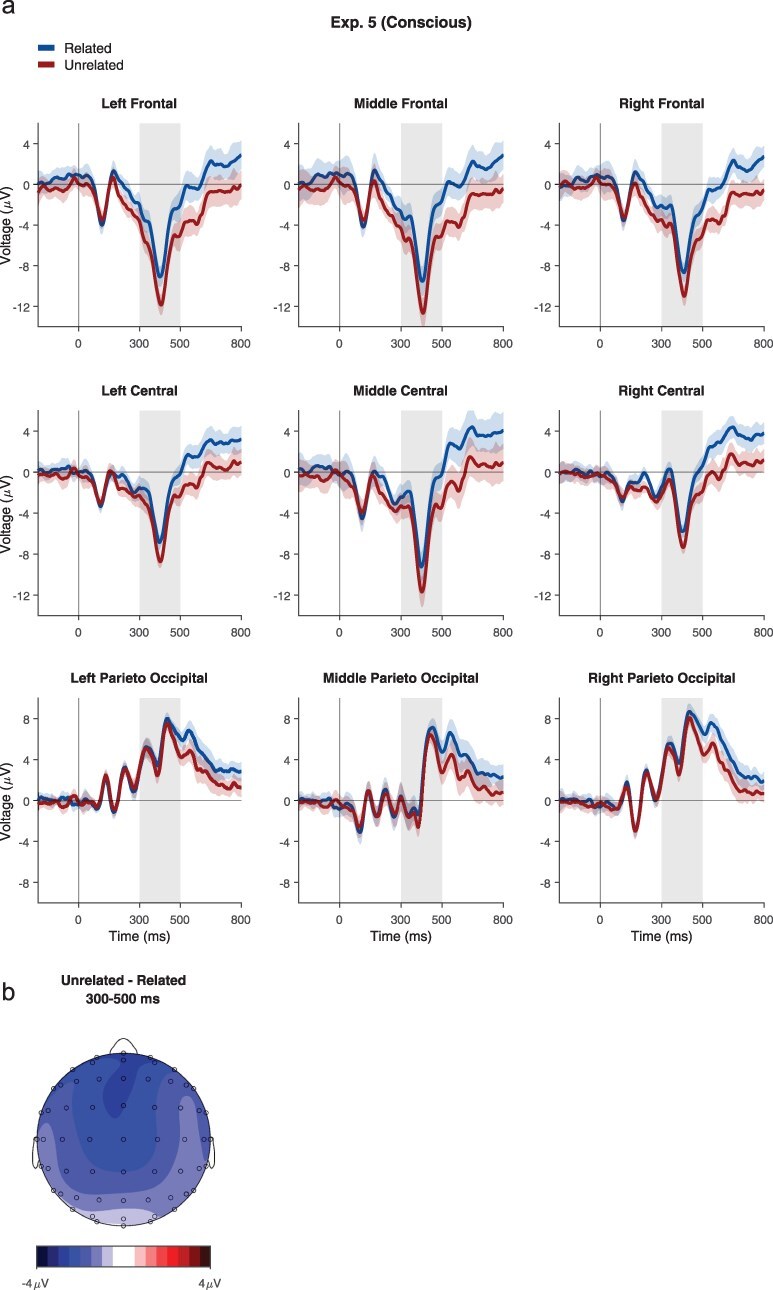
Results of Experiment 5. (a) Averaged waveforms from Experiment 5, locked to stimulus onset, for each condition and electrode group. The same conventions as in Figs 4 and 5 are used, but with darker colors, to denote conscious processing. (b) Topographic map of the effect of Relatedness (Unrelated–Related) at the N400 time window.

The behavioral results confirmed that this manipulation indeed allowed the pairs to be consciously processed: even with the short presentation duration, participants rated the vast majority of the trials as visible (visibility 1: 9 + 5.7%; visibility 2: 28.3 ± 10.5%; visibility 3: 27.1 ± 11.9%; visibility 4: 35.6 ± 12.3%) and were able to successfully classify the target pair as related/unrelated (*M* = 85.1%, SD = 10%). The EEG waveforms suggested an N400-like effect, with a widespread negativity that is frontocentrally oriented (Fig. 6). This was confirmed by a three-way ANOVA on ERP amplitudes yielding a main effect of relatedness (*F*(1, 11) = 17.34, *P* = .010, BF = 70.1), with an interaction between Relatedness and Region: (*F*(2, 22) = 6.94, *P* = .043, but BF = 0.21). *Post-hoc* tests revealed that the effect of Relatedness was stronger in the frontal and central electrodes (Frontal: *t*(11) = 4.06, *P* = .010, BF = 13.9; Central: *t*(11) = 3.77, *P* = .014, BF = 9; Paraieto-Occipital: *t*(11) = 2.57, *P* = .062, BF = 1.62). No other interactions were found (Relatedness and Laterality: *F*(2, 22) = 1.08, *P* = .50, BF = 0.1; Relatedness × Region × Laterality: *F*(4, 44) = 0.85, *P* = .54, BF = 0.06). Thus, even with the same stimuli and the same exposure duration used in Experiment 4, a clear N400 effect was found.

Again, combining Experiments 4 and 5 into one dataset showed significant differences between conscious and unconscious processing. We conducted a four-way ANOVA which included the experimental condition and found a significant two-way interaction between Experimental condition and Relatedness (*F*(1, 79) = 14.96, *P* = .002, BF = 6648). The other interactions of interest were all non-significant (Experiment × relatedness × Region: *F*(2, 158) = 2.27, *P* = .26, BF = 0.18; Experiment × Relatedness × Laterality: *F*(2, 158) = 0.81, *P* = .54, BF = 0.07; Experiment × Relatedness × Region × Laterality: *F*(4, 316) = 0.92, *P* = .54, BF = 0.03).

#### Decoding analysis

**Figure 7:**
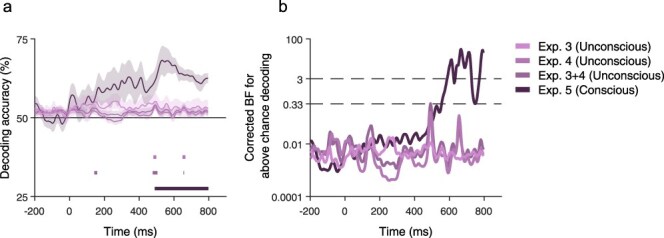
Decoding of Relatedness in Conscious vs. Unconscious conditions. (a) Decoder accuracy across time for the three EEG experiments (mean ± SEM). Light purple: Unconscious (Exp. 3); Medium purple: Unconscious (Exp. 4); Medium-dark purple: Unconscious (Exp. 3 + 4); Dark purple: Conscious (Exp. 5). For binary classification, the chance level is 50%. Horizontal lines denote time points with significant above chance decoding (*P* < .05, FDR corrected). (b) Corrected BF (in log scale) for decoding across time. In agreement with the frequentist analysis, decoding was above the BF = 3 threshold (top dashed line) for the conscious condition (Experiment 5) and below the BF = 0.33 (bottom dashed line) threshold for the unconscious conditions (Experiments 3–4). The frequentist and Bayesian analyses agree also when combining the two unconscious conditions together (Exp. 3 + 4).

To further assess the strength of our claim for no processing of relations without awareness, we tested if evidence for such processing might be unraveled using decoding, suggested to be more sensitive in finding effects that might not be picked up by the more traditional univariate analysis (Bae and Luck 2018). In addition, this analysis allowed us to more exploratory look for potential effects outside our initial temporal window of interest, as it was conducted on all time points. For Experiment 5 (conscious processing), robust above-chance decoding was found in the conscious condition, as expected, starting from ∼500 ms. Yet for Experiments 3 and 4 (unconscious processing), decoding did not differentiate between related and unrelated pairs. This further substantiates the conclusion that consciousness might indeed be needed for processing the relations between two simultaneously presented images. The results of this analysis are depicted in Fig. 7.

## Discussion

In the last decade or so, experimental efforts have been made to understand the role of consciousness in information integration (e.g. Van Opstal et al. 2010, Sklar et al. 2012, van Gaal et al. 2014, Faivre et al. 2019, Van Opstal and Rooyakkers 2022; for a recent review, see Hirschhorn et al. 2020). Step by step, different integrative processes have been probed using various techniques, asking if they can be observed in the absence of awareness. Unfortunately, many of these studies did not include a direct comparison with conscious processing. Their results sometimes conflicted, and the way awareness was measured and manipulated varied considerably between studies, making it harder to combine the findings into a coherent framework (for reviews, see Kouider and Dehaene 2007, Mudrik and Deouell 2022). Here, we conducted a systematic investigation of the role of consciousness in deciphering the relations between object pairs, held to require some form of integration (Mudrik et al. 2014), while carefully measuring awareness both at the subjective and the objective levels. Using two different methodologies—looking both for behavioral priming and for a neural signature of processing (namely, N400), we found clear effects during conscious processing, but not during unconscious processing.

In the conscious experiments, a behavioral priming effect and an EEG N400 effect were found. As expected, participants were faster to classify pairs that were preceded by another pair portraying the same relations, compared to pairs preceded by a pair portraying the opposite relations (i.e., a related pair preceded by an unrelated pair or vice versa). And, again in line with our hypotheses, unrelated pairs elicited a more negative N400 effect compared with related pairs. The difference between related and unrelated pairs was further demonstrated using decoding; when SVM models were trained and tested on the data from the conscious experiment, the relation type was clearly predicted by the EEG signal. These results are interesting irrespective of the question of consciousness; despite the simultaneous presentation of two distinct objects, even a relatively short presentation duration of 30 ms suffices to evoke clear detection of the relations between objects within a pair. This goes beyond previous studies, which either used much longer presentation durations (e.g. 500 ms, Zucker and Mudrik 2019) or presented the stimuli sequentially rather than simultaneously (Barrett and Rugg 1990, McPherson and Holcomb 1999).

In sharp contrast to conscious processing, four experiments provided strong evidence against unconscious processing of these relations: no effect was found when we tried to replicate the result in a preregistered experiment (this was also the case when the data from the two experiments were combined). Stronger evidence against such processing was obtained in the EEG experiments, where no difference was found between the conditions (in fact, in the more powered Experiment 4, the waveforms were practically identical for related and unrelated object pairs). This null result was further strengthened by the lack of decoding of relation type during unconscious perception despite the arguably higher sensitivity of the decoding method compared with the contrastive one (Bae and Luck 2018). Thus, when all results are taken together, the data suggest a dichotomous answer in the context of our experiments, whereby processing the relations between the objects took place with, but not without, awareness.

This evidence against unconscious processing is in line with several theories of consciousness, assigning a role for consciousness in integration (Lamme and Roelfsema 2000, Dehaene and Naccache 2001), or claiming that consciousness amounts to integrated information (Tononi et al. 2016, though note that IIT makes no predictions about the cognitive function of consciousness). It is also in line with a previous study showing that attention is needed to grasp the spatial relations between objects (Gronau and Shachar 2014). There, an advantage in the recognition of spatially consistent pairs of objects was found compared to inconsistent ones, yet this effect was eliminated once one of the objects in the pair was unattended. One could claim, on the other hand, that the findings do not align with the differential suppression times of spatially consistent vs. inconsistent pairs under CFS (Stein et al. 2015). However, breaking-CFS results are by and large no longer taken as evidence for unconscious processing, as they could also stem from post-perceptual processes (Stein et al. 2011). Furthermore, it is possible that in that study, the spatially consistent alignment of the two objects led to a more perceptual grouping, where they were processed as a singular complex object, thus not involving processing the associative relations between two distinct objects which were probed in our study. Under this assumption, then, the reported differential emergence times might have stemmed from the difference between processing a gestalt-like object, based on the perceptual grouping of the consistent pair, and processing two separate objects in the inconsistent pair.

Notably, however, it might be that in the current experiments, relation processing failed due to the spatial window over which integration had to take place, given the size of the presented stimuli. A recent study showed that unconscious integration of letters was possible, but only when the spatial window was small (Van Opstal and Rooyakkers 2022), in line with the “windows of integration” hypothesis (Mudrik et al. 2014, Hirschhorn et al. 2020). As the current study did not manipulate the size of the spatial window, we cannot exclude this interpretation, which should be tested in future studies.

Yet the results can also stem from unconscious processes being more limited than suggested above, failing to afford more basic processes than relation processing or integration. First, the obtained null results might simply stem from a failure to process two distinct stimuli without awareness, irrespective of integration. Thus far, the vast majority of studies probing unconscious processing have focused on the processing of single stimuli (e.g., a word or an isolated object; for review see, Kouider and Dehaene 2007, Mudrik and Deouell 2022). Hence, it could be that the problem does not lie in the attempt to process the relations between the stimuli, but in the need to simultaneously process two stimuli that are presented on screen at the same time. Indeed, studies have robustly demonstrated that when objects are simultaneously presented (during conscious processing), they compete with each other, evoking activations that are substantially reduced in the form of a weighted average between the activations evoked by each of the stimuli when presented in isolation (Kastner et al. 1998, MacEvoy and Epstein 2009). This further leads to a reduced ability to retrieve semantic information based on their visual features (Scalf et al. 2013) and decreased behavioral performance (Desimone and Duncan 1995). Performance is expected to be even worse during unconscious processing, where the stimuli are inherently degraded, and the signals notoriously weak to begin with (Greenwald et al. 1996).

At an even more basic level, the results might reflect an inability to process the stimuli themselves, even in isolation. Under this interpretation, our results join the line of studies that failed to find evidence for unconscious processing of objects (Bisiach and Rusconi 1990, Stein et al. 2020), as opposed to those that did report positive results, using either neuroimaging and neuropsychology (Marshall and Halligan 1988, McGlinchey-Berroth et al. 1993), or behavioral methods (Dell’Acqua and Grainger 1999, Fang and He 2005, Van den Bussche et al. 2009a).

Finally, another possible explanation for the results might stem from the fact that the stimuli in our experiment were all novel to the participants; no training or previous exposure took place prior to the experimental session, and other stimuli were used in the calibration session. This was aimed at preventing stimulus–response associations (Damian 2001), making sure that the observed effects—had they been found—truly represent relation processing. Notably, some previous studies chose to first train their participants with the stimuli, such that they first viewed them consciously (e.g. Sklar et al. 2012, van Gaal et al. 2014). Similarly, one study directly showed that the obtained effects—there, multisensory integration—are contingent upon previous conscious exposure (Faivre et al. 2014). Along the same lines, a meta-analysis reported stronger effects for familiar as opposed to novel primes (Van den Bussche et al. 2009b). Thus, the failure to obtain an effect might stem from this factor in our design, and future studies could revisit this question while first consciously exposing the participants to the stimuli.

Future studies are needed to determine the level at which unconscious processing fails; novel stimuli processing, single object processing, simultaneous processing, integration over large spatial windows, or integration irrespective of the window. Yet whichever the level at which unconscious processing stops, the results still tip the scales in the debate around the depth of unconscious processing toward a more restricted scope, challenging views that consider it to be all-encompassing (Hassin 2013). Indeed, skeptical views about unconscious processing have been raised since the inception of the field (Eriksen 1960). In recent years, in part thanks to the growing ability to publish null results and not only positive ones (Baxter and Burwell 2017), these views have been strengthened by empirical findings. In the context of integration, such studies either failed to reproduce earlier results across various stimulus types (e.g., words, scenes; Moors et al. 2016, Biderman and Mudrik 2017, Rabagliati et al. 2018) or demonstrated that integration can be performed between a visible and an invisible stimulus, but not between two invisible stimuli (Mongelli et al. 2019). Our findings thus align with these studies, and others suggesting a limited scope of unconscious processing (see again Stein et al. 2020, Van Opstal and Rooyakkers 2022, implying that consciousness might indeed enable some cognitive functions that cannot take place in its absence).

Several limitations should be considered when evaluating the results of this study and their implications. First, as our claims are based on null results in the unconscious condition, they should be taken with caution (because, clearly, absence of evidence does not entail evidence of absence; Alderson 2004). Our conclusions could have been stronger if we could demonstrate that some processing occurred during the unconscious condition, contrasting the failure to integrate not only with the conscious condition but also with a positive outcome in the unconscious one (see, for example, Biderman and Mudrik 2017, where such dissociation was found). Future studies could aim to find such dissociations, in line with our suggestion above to investigate the stage at which unconscious processing stops. Another potential avenue for future studies is to manipulate awareness using a within-participants design (i.e., by showing both masked and unmasked stimuli to the same participants). We opted against this design for two main reasons, both related to the limited number of stimuli we had. Specifically, a within-subject design would have required repeating the stimuli, and we were concerned that such repetition might evoke weaker effects on the one hand (Zucker and Mudrik 2019) and create response–stimulus associations that could confound the results on the other hand (Damian 2001). To explore the option of within-subject effects, we conducted additional analyses on our data, using the PAS to classify trials (see Supplementary Information - Extended Data 6); though notably, we had relatively few conscious trials in the unconscious condition, and vice versa.

Second, in the priming experiments, we observed differences in participants’ reaction times between the conscious and unconscious conditions (the latter being slower), and—due to the gradual introduction of the target pairs—it remains unclear when exactly they broke into awareness in these experiments. Presumably, if the difference between the prime and the target onsets was too long, priming effects might have been missed, as unconscious priming is known to decay quickly (Greenwald et al. 1996). We addressed this concern by analyzing only the faster half of the trials in Experiment 2, as the slower trials could have potentially obscured any priming effects observed in the faster trials, in line with previous studies suggesting that unconscious effects are more easily detected in such trials (Kinoshita and Hunt 2008, Ansorge et al. 2010, Avneon and Lamy 2018, Baumann and Valuch 2022). Yet in this analysis as well, null results were found. Additionally, the EEG experiments were explicitly designed to mitigate such concerns (i.e., that the pairs were processed unconsciously but failed to subsequently evoke priming). There, we again found no evidence of such processing in the unconscious condition (as opposed to the conscious one).

A third limitation pertains to the number of trials per condition in this study, which might have been too low to observe small unconscious effects. This number was limited mainly by two factors: the number of stimuli we had (with a preference to avoid repetitions as explained above) and the length of the experiments, as we aimed to prevent very long sessions that might reduce participants’ engagement. Notwithstanding this limitation, we argue that it is less likely to account for the absence of an effect in the unconscious conditions for three reasons: First, in Experiment 4, we chose to both increase the number of trials and nearly double the number of participants to enhance statistical power, yet the results remained unchanged. Second, regarding the decoding analysis, we note that the number of trials per condition in the EEG experiments was comparable to the original study that informed our decoding approach (Bae and Luck 2018), and that even when we combined the data from Experiments 3 and 4, we could not detect such an effect.

The final limitation of this study stems from the fact that participants always responded to the pairs with the same fingers (counterbalancing was done across participants, but not within participant). Potentially, the significant decoding in the conscious condition could reflect decoding of the response finger rather than the representation of relatedness (as in this condition, accuracy was high, so the two factors are correlated). This potential confound cannot be overruled, though we note that decoding motor acts with scalp EEG is typically done at the hand level rather than the finger level, so more research is needed to determine how likely this confound is to explain the results. Moreover, since we found clear differences in both priming and neural activity in the univariate analysis, it seems more plausible that there was indeed some representation of relatedness in the conscious condition, which was picked up by the decoding analyses.

In conclusion, the reported set of experiments represents a systematic and rigorous attempt to explore the limits and scope of unconscious integration, specifically targeting the ability to process the relations between two objects. Using both behavioral methods and neural recordings, and including a direct comparison between conscious and unconscious processing, we showed that the latter is rather limited. We took different measures to give the unconscious effect the best chance to be found: we turned to electrophysiology, held to be more sensitive than behavioral measures (Luck et al. 1996) after the behavioral effect failed to replicate. When we still found no evidence for unconscious processing—alongside clear effects for conscious one—we tried a “lighter” masking procedure (Breitmeyer 2015) and appealed to decoding, held to be more prone to detect weaker effects that might be missed by the typical univariate analysis (Bae and Luck 2018). Complementing those measures with Bayesian analysis, which by and large supported the frequentist null results, and following predefined and preregistered protocols, we argue that our approach yielded compelling evidence against unconscious processing of the relations conveyed by object pairs. This does not mean that evidence for such relation processing would not be found in the future, with better experimental procedures or analysis approaches, as is true for every null result (Alderson 2004). Yet given the current state of affairs, we conclude that it seems reasonable to endow a prominent role for consciousness in enabling integration beyond the single object level and potentially also in more basic processes, including the identification of real-life objects.

## Supplementary Material

niaf010_Supp

## Data Availability

Raw and analyzed data of this study can be found in the following link: https://osf.io/ewykz/?view_only=78f6f6a8f8594a038375394880a25d72.
