# Supplementary material for: Object relations are processed with, but not without, awareness
Source: Neurosci Conscious. 2025 May 8;2025(1):niaf010. doi: 10.1093/nc/niaf010 (PMC12063529; doi:10.1093/nc/niaf010)
Supplement: niaf010_Supp [file niaf010_supp.zip › suppl_data/PalgiBester_Supplementary-Information_clean.docx]

# Supplementary Materials

# **Extended Data 1: List of exclusion criteria**

The criteria for participants' exclusion were: (a) target performance below 70%, (b) average reaction time deviating by two standard deviations from the overall group average, and (c) fewer than 15 valid trials in any experimental condition (Repetition/Non-repetition ✕ Related/Unrelated, for a total of 4 conditions) in the behavioral experiments, and fewer than least 20 valid trials in each condition (Related/Unrelated), in the EEG experiments, after removing eye movement or muscular artifacts.

Participants who met any individual criterion for exclusion were replaced until sample size was complete. All of the criteria were established prior to the data collection stage, during experiment planning and pre-registration (when applicable).

**Extended Data 2: Stimuli of post-session visibility test**

In addition to the object-pairs used in the main session, another set of pairs (all unrelated) was used for the post-session visibility test, and for the calibration session (see Procedure). In experiments 2 and 3, 200 pairs were used, where half of them were taken from the previously described pool^1^, and half were new unrelated pairs we added. In experiment 4, all images in these two sessions were new, either taken from the Bank of Standardized Stimuli N= 386 images; ^2^ or created by us (N=24), collected from various internet sources. Since the behavioral task in the calibration and the post-visibility sessions refers to the orientation of the objects in the pair, half of the images were digitally rotated so that the object presented in them would face upwards, thereby making its orientation unambiguous. Images were randomly divided into pairs, so that no image was repeated between pairs, and were divided into blocks so that one exemplar appeared in each block (that is, in a block, each object appeared only once). In all experiments, these images were presented on a gray background, akin to the main experimental session. As we used only unrelated object pairs for the post-session visibility test and for the calibration session, we validated that object relations do not influence the visibility of the target. We compared the proportion of trials where participants reported that they did not see anything (‘visibility 1’ trials) for related and unrelated pairs in the main session. No difference was found (Related: 79.6±17.3% of the trials classified as not seen, Unrelated: 79.9±17.2% of the trials classified as not seen; t(24)=-0.36, p=0.72), suggesting object relations did not influence the pairs visibility.

**Extended Data 3: Excluding participants with slow reaction times**

In Experiment 1, two participants were slower to respond to the targets as compared to the other 10 participants (mean reaction times of these two participants were >2800 ms, reaction time for all other participants was 1522±388 ms, mean±SD). This resulted in the rejection of more than 10% of their trials due to too slow responses (RT>4 s). To make sure this high exclusion did not influence the results, we re-analyzed the data of Experiment 1 after discarding these two participants.

Akin to the main analysis, we found a significant main effect for Congruency (F(1,2426)=82.9, p<0.001, BF>1015), and a significant interaction between Congruency and Relations (F(1,2426)=15.7, p<0.001, 95%CI=[-237 -154], BF=119). Here again, the effect was stronger for related pairs (M_diff_=200ms, SD=83ms, t(9)=7.6, p<0.001, 95%CI=[-259 -140], BF=721) than for unrelated pairs (M_diff_=80ms, SD=100ms, t(9)=2.5, p=0.035, 95%CI=[-153 -7], BF=2.3).

**Extended Data 4: Results of Pilot Experiment**

The procedure of the pilot experiment was identical to the pre-registered Experiment 2 described above. Some evidence for unconscious priming was indeed found, albeit weak (see Extended Figure 2-2 below), motivating the replication attempt (where we took a Bayesian approach in an attempt to find conclusive evidence).

Here, the overall performance for the visible target was 89.3% (SD=5.7%). 73% of all trials (SD=24.3%) were rated as completely invisible, and all analyses were done only on these trials. Performance was at chance for the relatedness of prime pairs (M=51.5%, SD=3.1%; M d’=0.082, SD=0.22; t(23)=1.86, p=0.08, 95%CI=[-0.009 0.17], BF=0.94). Notably, above chance performance was found for the orientation task, in the post-session visibility test, though with no conclusive evidence in the Bayesian analysis (M=53.1%, SD=12.1%; M d’=0.33, SD=0.61; t(19)= 2.42, p=0.026, 95%CI=[0.045 0.62], BF=2.36; note though that in both objective measures, the effects were marginally significant, possibly suggesting a trend towards above chance performance). Yet even though participants were unaware of the prime pair, priming was still found, indicated by a main effect of prime-target congruency, though this was not confirmed by the Bayesian analysis (F(1,4383)=7.46, p=0.025, 95%CI=[-77 -20], BF=0.93): Like in Experiment 1, participants were faster in congruent (M =1433ms, SD=249ms) than in incongruent trials (M=1460ms, SD=249ms). This time, Prime-target congruency and target relations only marginally interacted, again with no support from the Bayesian analysis (F(1,4382)=4.38, p=0.079, BF=0.35): here, the effect was found only for related (M =43ms, SD=71ms, t(23)=2.98, p=0.025, 95%CI=[-73 -13], BF=6.8) but not for unrelated pairs (M=12ms, SD=59ms; t(23)=1.02, p=0.46, 95%CI=[-37 13], BF=0.34). Thus, the results seem to hint towards an unconscious priming effect; to test the robustness of this effect, Experiment 2 was conducted.


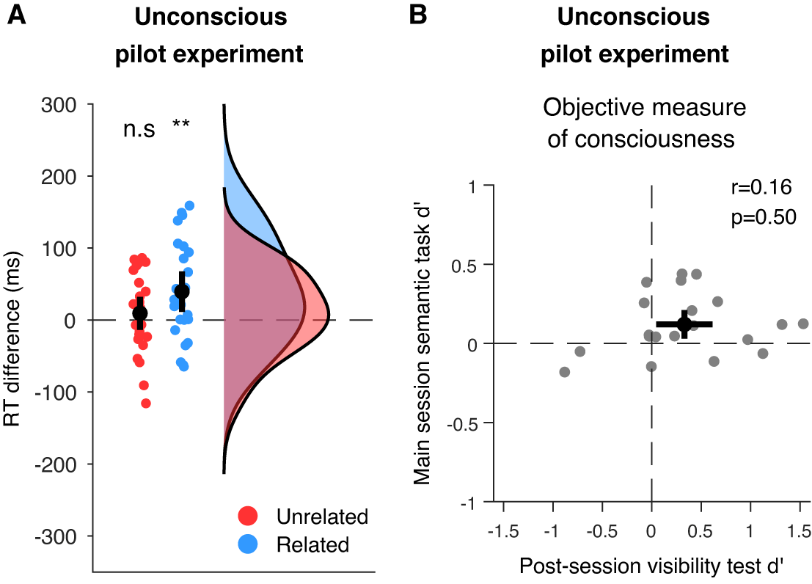


**Supplementary Fig. 1: Pilot experiment results:** Results of the pilot experiment we conducted, testing unconscious processing. Mean priming effect, calculated by subtracting reaction times (RTs) in repetition trials from non-repetition trials. Data is plotted separately for Related targets (red) and Unrelated targets (blue). Dots correspond to individual participants (n=24), with black dots and lines showing the population means and CIs. **B.** Objective measure of consciousness. Individual participant d’ values (n=20) for the main session semantic task (Y-axis) vs. the post-session visibility test (X-axis). Black dots and lines correspond to the population means and CIs. Fewer participants are plotted due to exclusion from the post-test visibility session.

**Extended Data 5: analysis of participants accuracy**

To complement the main analyses reported in the manuscript, we describe here the results of analyzing participants’ accuracy in the main session of each experiment.

**Experiments 1 & 2 (Priming):**

In these two experiments, we asked whether prime-target congruency affected participants’ accuracy. A mixed-effects logistic regression with the same formula as the one used for reaction time, fitted using maximum likelihood estimation, did not reveal any such effect, both in the conscious condition (Exp. 1; Congruency: z=0.96, p=0.34; Congruency X Target relation: z=-0.84, p=0.40), and in the unconscious condition (Exp. 2; Congruency: z=0.28, p=0.78; Congruency X Target relation: z=0.64, p=0.52).

**Experiments 3-5 (EEG):**

Here, we tested the effect of visibility (subjectively assessed using the PAS ratings) on participants’ accuracy. This allowed us to validate the PAS in our experiments, as we expected that objective performance would improve with greater visibility. We evaluated this using a mixed-effects logistic regression with accuracy as the dependent variable, visibility (seen / unseen) and object relations as the independent variables, and participant as a random variable. Indeed, participants’ accuracy was higher in trials deemed visible (Exp. 3; Visibility: t(3702)=7.6, p<10^-13^; Visibility X Relations: t(3702)=-7.48, p<10^-13^; Exp. 4; Visibility: t(7541)=3.4, p=0.0006; Visibility X Relations: t(7541)=-2.47, p=0.014; Exp. 5: Visibility: t(1879)=9.2, p<10^-19^; Visibility X Relations: t(1879)=-7.7, p<10^-13^).


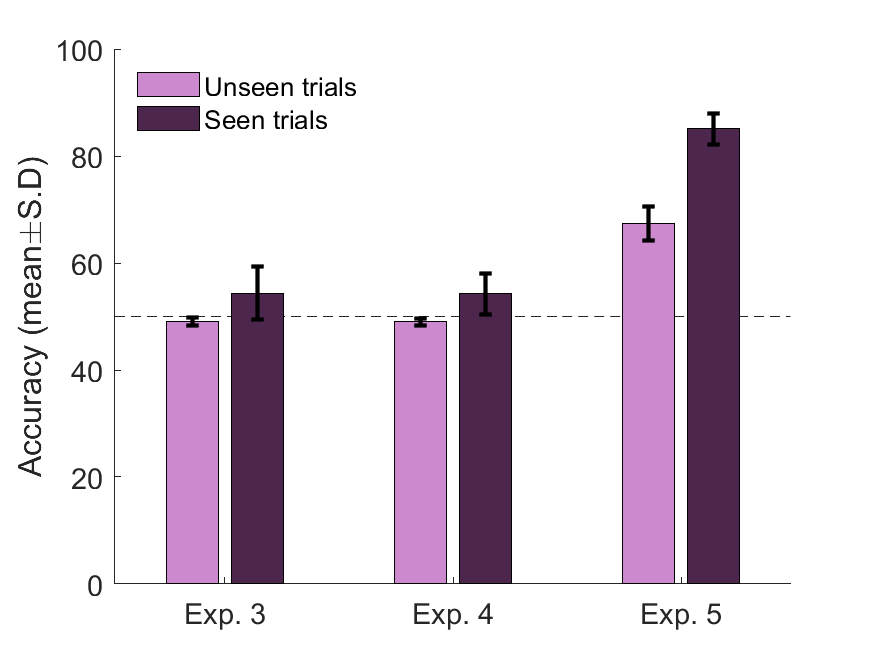
**Supplementary Fig. 2: Accuracy scores for the two visibility conditions, in the three EEG experiments.** The results of this analysis confirm the correspondence between the objective measure (trial accuracy) and the subjective measure (PAS score). We note that this correspondence is far from perfect: in Experiments 3 & 4 accuracy is low in the seen trials, while in Experiment 5, above chance accuracy is found for unseen trials. This discrepancy probably stems from the limited number of trials in these conditions (as there were not many seen trials in Experiment 3 & 4, and not many unseen trials in Experiment 5: [Exp. 3: 14±4 visible trials per participant, for 18 out of 24 participants; the others had no visible trials whatsoever; Exp. 4: 30±4 visible trials per participant, for 36 out of 45 participants; Exp. 5: 20±4 invisible trials for the 12 participants]). Crucially, we used the PAS mainly to assess trial invisibility in the unconscious conditions (Exp. 3 & 4), and trial visibility in the conscious condition (Exp. 5) – in these cases, where the number of trials is adequate, we see chance-performance for unseen trials, and very high performance on seen trials.

**Extended Data 6: Within-participants analysis**

Our experimental design did not manipulate awareness within participants, but rather between experiments. Nevertheless, as participants had some invisible trials in the conscious conditions (Exp. 1 & 5), and some visible trials in the conscious conditions (Exp. 2-4), we could theoretically examine the effects of awareness in a within-participants design. We note again though that this analysis is limited due to the low number of trials in these conditions.

**Experiments 1-2 (Priming):**

In Experiment 1 participants reported the target pairs as visible in 98.9% of the trials, leaving very few invisible trials. No single participant passed our inclusion criterion of having a minimum of 15 trials per experimental condition, and only three participants even had *any* trials in all experimental conditions. This very small number of trials did not allow us to analyze Experiment 1 in this manner.

In Experiment 2, participants reported the pairs as invisible in 79.7% of the trials, leaving slightly more visible trials per participant. Three participants passed our inclusion criteria of having at least 15 trials per condition, and 15 participants had at least one trial in all experimental conditions. We used a similar linear mixed-effects model as before, adding Visibility (Visible: PAS 3-4, Invisible: PAS 1) as another fixed factor. This model did not suggest an interaction between Visibility and Congruency (F(1,5388)=0.21, p=0.65, BF=0.13), nor a triple interaction between Visibility, Congruency and Relations (F(1,5382)=0.71, p=0.19, BF=0.29).

**Experiments 3-5 (EEG):**

In all three EEG experiments, there was a very low number of trials in the opposite condition: a low number of visible trials in each experimental condition in Experiments 3 (M=4.1, SD=6.8) and 4 (M=9.6, SD=11.1), and similarly, a low number of invisible trials in each experimental condition in Experiment 5 (M=6.3, SD=4.8). Adopting our inclusion criterion of at least 20 trials in each condition, very few participants could be included, even before further trial exclusion due to artifact removal: two participants in Experiment 3, ten in Experiment 4, and none in Experiment 5. Thus, we could not examine the ERP patterns using a within-participant design.

**Extended Data 7: Linear Mixed Models (LMMs) analysis of the EEG data**

Above we report the results analyzed using a 3-way ANOVA, in line with previous works from our lab^1,3^, and with the pre-registered analysis plan. Yet to make sure we are not missing any effect due to that choice, we also ran the same analysis with Linear Mixed Models. The EEG data was averaged in the N400 window (300-500 ms) and was grouped like in the ANOVA analysis. The main difference is that here we used single trials and not condition-averaged data. Fitting of the models was done similarly to the priming analysis, both for the Frequentist and Bayesian approaches. All models included a per-subject random slope for Relatedness^4^. The results for the three experiments were as follows:

**Exp. 3 (Unconscious):**

No main effect of relatedness was found (F(1,23)=1.51, p=0.48, BF=0.09). The 2-way interaction between relatedness and laterality was also non-significant (F(2, 25316)=0.0003, p>0.99, BF=0.001), and also the 3-way interaction between relatedness, region and laterality (F(4, 25316)=0.32, p>0.99, BF<10^-4^). In this analysis, however, we did find a significant interaction between relatedness and region, though it was not corroborated by the Bayesian results (F(2, 25316)=5.47, p=0.02, but BF=0.22). Importantly, post-hoc tests looking for the source of this interaction did not yield significant results in any electrode group (Frontal: F(1,24)=1.99, p=0.41, BF=0.07; Central: F(1,21)=1.47, p=0.48, BF=0.059; Paraieto-Occipital: F(1,23)=0.06, p>0.99, BF=0.059).

**Exp. 4 (Unconscious):**

Here, no significant effects were found: not a main effect of relatedness (F(1,43)=0.12, p>0.99, BF=0.044), nor any of the interactions (relatedness × region: F(2, 53965)=0.84, p=0.69, BF=0.001; relatedness × laterality: F(2, 53965)=0.1, p>0.99, BF<10^-3^; relatedness × region × laterality: F(4, 53965)=0.18, p>0.99, BF<10^-5^).

**Exp. 5 (Conscious):**

The results were in line with the ANOVA findings; a main effect of relatedness was found (F(1,11)=7.97, p=0.049, but BF=0.31), as well as an interaction between relatedness and region (F(2,11777)=14.83, p<10^-5^, BF>10^3^). The other interactions were not significant (relatedness × laterality: F(2, 11777)=0.43, p=0.98, BF=0.003; relatedness × region × laterality: F(4, 11777)=0.17, p>0.99, BF<10^-3^). Post-hoc tests for the significant interaction showed an effect only for the frontal and central electrodes (Frontal: F(1,10)=13.1, p=0.02, BF=0.23; Central: F(1,11)=7.84, p=0.049, BF=0.19; Parieto-Occipital: F(1,11)=1.05, p=0.58, BF=0.1).

**Extended Data 8: discussion for Experiment 4**

In Experiment 4, where the evidence against an unconscious effect was the strongest, the participants were actually able to discriminate above chance – albeit very slightly – between the related and the unrelated pairs, as reflected by their sensitivity measure (d’) which was significantly higher than 0 at the group level. Such above-chance performance might be expected, as we selected trials for analysis based on subjective invisibility, i.e. the PAS ratings^5^. Indeed, a recent study showed that the threshold for subjective invisibility is higher than that of objective invisibility, which is in line with our results here^6^. Intriguingly though, this above-chance performance was not found for the arguably easier, non-semantic task, which was taken in the post-experiment visibility test. A possible explanation for these results is that participants might have performed poorly in the post-experimental session due to fatigue or lack of motivation^7,8^. Importantly though, even if this above-chance performance indicates that participants might have been aware of the pairs in some of the trials or nevertheless pushed towards a correct response due to unconscious processing; ^9^, this was not reflected in any way in the resulting ERPs. However, they do highlight the difficulty of appropriately measuring awareness, an open issue that is still widely discussed in the field^9-12^.

**References**

1 Zucker, L. & Mudrik, L. Understanding associative vs. abstract pictorial relations: An ERP study. *Neuropsychologia* **133**, 107127 (2019).

2 Brodeur, M. B., Guérard, K. & Bouras, M. Bank of Standardized Stimuli (BOSS) Phase II: 930 New Normative Photos. *PLOS ONE* **9**, e106953 (2014). https://doi.org:10.1371/journal.pone.0106953

3 Truman, A. & Mudrik, L. Are incongruent objects harder to identify? The functional significance of the N300 component. *Neuropsychologia* **117**, 222-232 (2018). https://doi.org:https://doi.org/10.1016/j.neuropsychologia.2018.06.004

4 Oberauer, K. The Importance of Random Slopes in Mixed Models for Bayesian Hypothesis Testing. *Psychological Science* **33**, 648-665 (2022). https://doi.org:10.1177/09567976211046884

5 Ramsøy, T. Z. & Overgaard, M. Introspection and subliminal perception. *Phenomenology and the Cognitive Sciences* **3**, 1-23 (2004).

6 Stein, T., Kaiser, D., Fahrenfort, J. J. & van Gaal, S. The human visual system differentially represents subjectively and objectively invisible stimuli. *PLOS Biology* **19**, e3001241 (2021). https://doi.org:10.1371/journal.pbio.3001241

7 Finkbeiner, M. Subliminal priming with nearly perfect performance in the prime-classification task. *Attention, Perception, & Psychophysics* **73**, 1255-1265 (2011). https://doi.org:10.3758/s13414-011-0088-8

8 Pratte, M. S. & Rouder, J. N. A task-difficulty artifact in subliminal priming. *Attention, Perception, & Psychophysics* **71**, 1276-1283 (2009). https://doi.org:10.3758/APP.71.6.1276

9 Sandberg, K., Timmermans, B., Overgaard, M. & Cleeremans, A. Measuring consciousness: Is one measure better than the other? *Consciousness and Cognition* **19**, 1069-1078 (2010). https://doi.org:https://doi.org/10.1016/j.concog.2009.12.013

10 Michel, M. How (not) to underestimate unconscious perception. *Mind & Language* **37** (2022). https://doi.org:https://doi.org/10.1111/mila.12406

11 Michel, M. The Mismeasure of Consciousness: A Problem of Coordination for the Perceptual Awareness Scale. *Philosophy of Science* **86**, 1239-1249 (2019). https://doi.org:10.1086/705509

12 Shanks, D. R. Regressive research: The pitfalls of post hoc data selection in the study of unconscious mental processes. *Psychonomic Bulletin & Review* **24**, 752-775 (2017).
