# Supplementary material for: Object relations are processed with, but not without, awareness
Source: Neurosci Conscious. 2025 May 8;2025(1):niaf010. doi: 10.1093/nc/niaf010 (PMC12063529; doi:10.1093/nc/niaf010)
Supplement: niaf010_Supp [file 1_cov.pdf › suppl_data/1_cov.pdf]

Prof. Liad Mudrik
Laboratory of high-level cognition

School of Psychological Sciences
and Sagol School of neuroscience

TEL AVIV UNIVERSITY אוניברסיטת תל אביב

Tel Aviv University
Haim Levanon 55
P.O. Box 39040
Tel Aviv
Phone: +972-3-6409143
mudrikli@tauex.tau.ac.i

April 12th, 2024

Dear editor,

We are pleased to submit our manuscript titled “Object relations are processed with, but not without, awareness” for potential publication as an article in *Neuroscience of Consciousness*. The potential role of consciousness in the integration of information has been a matter of ongoing debate, lying at the basis of several theories of consciousness. The literature has yielded mixed findings, with some papers suggesting that semantic integration can take place without awareness, and some claiming that it is limited to conscious processing. Only very few studies examine this question while comparing both conscious and unconscious processing, over a series of experiments, using both behavioral and electrophysiological measures.

Here, we do all that and provide strong and unequivocal evidence for the role of consciousness in information integration. Over five experiments (three of which preregistered), using both EEG and priming results, we show that processing of visual object relations take place with, but not without, awareness. This result is confirmed using Bayesian analysis, and demonstrated also using multivariate analyses of electroencephalographic signals. Taken together, our results substantially strengthen claims about the role of consciousness in information integration, and challenges arguments for the omnipotence of unconscious processes. All materials and code, as well as the preregistrations, are available on OSF.

We believe that the results will be of great interest to the wide readership of *Neuroscience of Consciousness*, and accordingly hope that you will find our manuscript suitable for review.

Sincerely,

Shaked Palgi, Tamara Bester, Nathan Faivre, and Liad Mudrik
